# Pleural effusion as the initial clinical presentation in disseminated cryptococcosis and fungaemia: an unusual manifestation and a literature review

**DOI:** 10.1186/s12879-015-1132-4

**Published:** 2015-09-22

**Authors:** Mayun Chen, Xiaomi Wang, Xianjuan Yu, Caijun Dai, Dunshun Chen, Chang Yu, Xiaomei Xu, Dan Yao, Li Yang, Yuping Li, Liangxing Wang, Xiaoying Huang

**Affiliations:** Division of Pulmonary Medicine, The First Affiliated Hospital of Wenzhou Medical University, Key Laboratory of Heart and Lung, Wenzhou, China; Wenzhou Medical University, Wenzhou, China; Division of Radiology Medicine, The First Affiliated Hospital of Wenzhou Medical University, Wenzhou, China

**Keywords:** Pleural effusion, Meningitis, Organ transplant, Pulmonary fungus, Immunocompromised, Cryptococcosis, Antifungal therapy

## Abstract

**Background:**

*Cryptococcus neoformans* infection usually presents as chronic meningitis and is increasingly being recognized in immunocompromised patients. Presentation with pleural effusion is rare in cryptococcal disease; in fact, only 4 cases of pleural effusion as the initial clinical presentation in cryptococcosis have been reported in English-language literature to date. We report the first case of pleural effusion as the initial clinical presentation in a renal transplant recipient who was initially misdiagnosed with tuberculous pleuritis but who then developed fungaemia and disseminated cryptococcosis. The examination of this rare manifestation and the accompanying literature review will contribute to increased recognition of the disease and a reduction in misdiagnoses.

**Case presentation:**

We describe a 63-year-old male renal transplant recipient on an immunosuppressive regimen who was admitted for left pleural effusion and fever. Cytological examinations and pleural fluid culture were nonspecific and negative. Thoracoscopy only found chronic, nonspecific inflammation with fibrosis in the pleura. After empirical anti-tuberculous therapy, the patient developed an elevated temperature, a severe headache and vomiting and fainted in the ward. Cryptococci were specifically found in the cerebrospinal fluid following lumbar puncture. Blood cultures were twice positive for *C. neoformans* one week later. He was transferred to the respiratory intensive care unit (RICU) immediately and was placed on non-invasive ventilation for respiratory failure for 2 days. He developed meningoencephalitis and fungaemia with *C. neoformans* during hospitalization. He was given amphotericin B liposome combined with 5-flucytosine and voriconazole for first 11 days, then amphotericin B liposome combined with 5-flucytosine sustained to 8 weeks, after that changed to fluconazole for maintenance. His condition improved after antifungal treatment, non-invasive ventilation and other support. Further pathological consultation and periodic acid-Schiff staining revealed Cryptococcus organisms in pleural sections, providing reliable evidence for cryptococcal pleuritis.

**Conclusion:**

Pleural effusion is an unusual manifestation of cryptococcosis. Cryptococcal infection must be considered in the case of patients on immunosuppressives, especially solid-organ transplant recipients, who present with pleural effusion, even if pleural fluid culture is negative. Close communication between the pathologist and the clinician, multiple special biopsy section stains and careful review are important and may contribute to decreasing misdiagnosis.

## Background

Cryptococcosis usually manifests as central nervous system (CNS) disease (meningoencephalitis) or pneumonia [1]. In particular, CNS disease is the most common Cryptococcus infection among late post-transplant recipients [2]. Pulmonary parenchymal lesions caused by cryptococcal infection, such as subpleural nodules, interstitial infiltrates, pulmonary masses, alveolar consolidation and lymphadenopathy, are occasionally encountered as well [3], whereas pleural effusion in rarely occurs cryptococcosis. To our knowledge, only 4 cases of pleural effusion as the initial clinical presentation in cryptococcosis have been reported in the English-language literature to date [4–7]. Here, we present the first case of disseminated cryptococcosis manifesting as meningitis, fungaemia and cryptococcal pleuritis with an initial clinical presentation of pleural effusion in a male kidney transplant recipient. The patient was initially misdiagnosed with tuberculous pleuritis. The review of the characteristics of this rare case and discussions related to diagnostic and management issues, in addition to a review of the relevant literature will contribute to recognition of the disease and a decrease in misdiagnoses.

## Case presentation

**Fig. 1:**
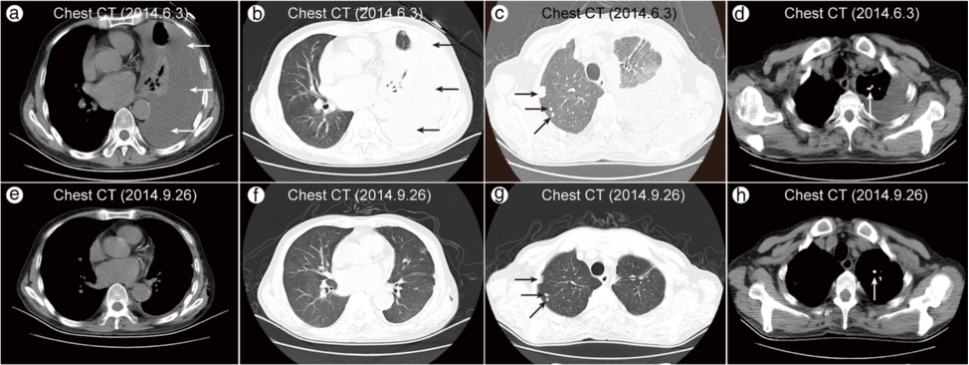
Chest CT (2014.6.3) showed a left-sided pleural effusion with compressive atelectasis of the left lower lobe (**a**, **b**), multiple nodules scattered in both lungs (**c**), and calcification in the nodules in the left upper lobe (**d**). Chest CT (2014.9.26) revealed that the left pleural effusion was completely resolved (**e**, **f**), but multiple nodules and calcification were still present (**g**, **h**)

A 63-year-old Chinese businessman, who had received a kidney transplant 17 months prior because of uraemia and who was being immunosuppressed with mycophenolate mofetil (MMF), methylprednisolone (MP) and tacrolimus (FK506), was admitted on June 3, 2014 (day 1) due to dyspnoea and fever lasting for two days. The patient denied having diabetes, hepatitis, or a history of contact with people diagnosed with tuberculosis (TB). Physical examination revealed a temperature of 38.5 °C and decreased breath sounds at the left base with dullness on percussion. A chest computed tomography (CT) scan showed left-sided pleural effusion, along with compressive atelectasis of the left lower lobe and scattered multiple nodules on both sides, and calcification was observed in the nodules in the left upper lobe (Fig. 1a–d). The blood count showed granulopaenia and lymphopaenia: white blood cell (WBC) count: 2870/μl, neutrophil (N) count: 1260/μl, lymphocyte (L) count: 540/μl, red blood cell (RBC) count: 4,620,000/μl, haemoglobin (HB): 133 g/L, and blood platelet (PLT) count: 170,000/μl.

Left thoracentesis was performed. The fluid was a slightly turbid yellow exudate and was negative for microorganisms on smear and culture (Table 1). The pleural effusion parameters were as follows: protein: 40.6 g/L, lactic dehydrogenase (LDH): 121 IU/L, WBC count: 920/μl with 70 % lymphocytes, RBC count: 2500/μl, adenosine deaminase (ADA): 121 IU/L, and glucose: 7.83 mmol/L (measured on June 4 (day 2)). All other laboratory examination results were negative: specifically, tumour markers were normal, the PPD-S skin test result was 0 mm, the sputum was negative for acid-fast bacilli, and sputum culture was negative.

**Table 1:** Parameters of pleural effusion

Date:	June 3	June 18
Colour:	yellow, slightly turbid	yellow, turbid
WBCs (/μl)	920	470
RBCs (/μl)	2500	5480
Lymphocytes (%)	70	98
Endotheliocytes (%)	30	-
Segmented cells (%)	-	1
Mononuclear macrophages (%)	-	1
Rivalta’s test	positive	positive
Total protein (g/L)	40.6	36.8
Lactate dehydrogenase (IU/L)	121	188
Glucose (mmol/L)	7.83	4.73
Adenosine deaminase (IU/L)	24	25
CEA (μg/L)	0.72	0.81
Culture:		
*Cryptococcus neoformans*	(−)	(−)
*M. tuberculosis*	(−)	(−)
Smear: 920		
*M. tuberculosis*	(−)	(−)

*WBC* white blood cell, *RBC* red blood cell, *CEA* carcinoembryonic antigen, *M. tuberculosis Mycobacterium tuberculosi*s

The patient was treated with intravenous cefoperazone + sulbactam and was kept on oral FK506, MMF and MP. His temperature dropped to normal the day after admission but rose three days later. Due to persistent granulopaenia and lymphopaenia (Table 2), he was treated with recombinant human granulocyte colony-stimulating factor (rhG-CSF) seven days after admission (day 8). A chest CT was repeated 7 days later, and the left pleural effusion was considerably decreased, but multiple nodules were still present. The patient was then transferred to the pulmonary division on June 12, 2014 (day 10).

**Table 2:** Peripheral blood cell count

Date:	June 4	June 5	June 10	June 12
WBCs (/μl)	2870	2320	1640	11190
Lymphocytes (/μl)	540	480	610	650
Neutrophils (/μl)	1730	1260	350	9630

*WBC* white blood cell

On admission to the pulmonary division, the patient’s body temperature was 38.1 °C, and lung examination revealed decreased breath sounds at the left base, with dullness on percussion. Laboratory test results included the following: the erythrocyte sedimentation rate (ESR) was elevated, to 60 mm/h; C-reactive protein (CRP) was elevated, to 116 mg/L; the haematocrit was 37 % peripheral; haemoglobin was 121 g/L; and the WBC count was 11,190/μl, with absolute lymphocytes at 650/μl, 86.1 % polymorphs and 5.8 % lymphocytes. Antinuclear antigen (ANA), extractable nuclear antigen (ENA), antineutrophil cytoplasmic antibody (ANCA), procalcitonin (PCT), arterial blood gas (ABG), blood tumour markers, and liver and renal functions were all within the normal ranges. Additionally, the sputum was negative for acid-fast bacilli and sputum culture, and interferon gamma release assay the (IGRA)/T-SPOT.TB were both negative.

To evaluate the pleural effusion, another thoracentesis was performed, and the pleural fluid was still a slightly turbid yellow exudate lacking organisms and malignant cells (Table 1), with the following laboratory results: protein: 36.8 g/L, glucose: 4.73 IU/L, LDH: 188 IU/L, and cell count: 470/μl (1 % N, 98 % L, 1 % Macrophage). The cytology of the pleural fluid was nonspecific. The lymphocyte predominance in the pleural effusion exudate prompted us to test for TB AND malignancy as possible causes of the pleural effusion. The ADA level in the fluid was 25 IU/L, and a polymerase chain reaction (PCR) analysis of the fluid for TB was negative. Moreover, the tumour markers in the fluid were all normal, and cultures of the pleural fluid for bacterial organisms, mycobacteria, and fungi were all negative.

**Fig. 2:**
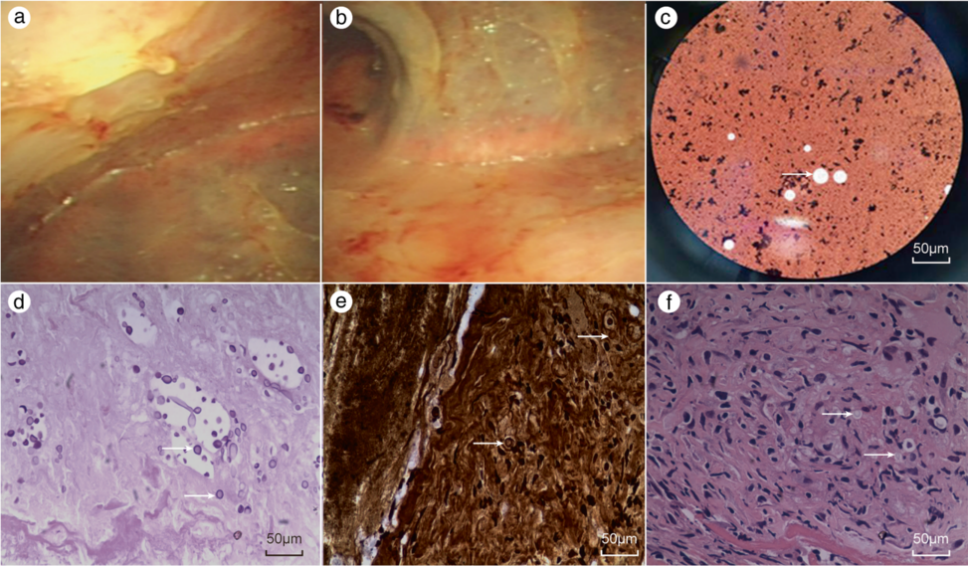
Intuitive thoracoscopy revealed localized pleural adhesion and a diffuse, cellulose-like pleura (**a**, **b**). *Cryptococcus neoformans* was identified by direct India ink staining of the cerebrospinal fluid (CSF) (**c**). Periodic acid-Schiff stain (PAS) and methenamine silver stain revealed numerous Cryptococcus organisms in pleural sections (**d**, **e**). Re-examination of previous sections revealed Cryptococcus-like organisms that had been previously overlooked, and the capsule was not stained (**f**)

A visualized thoracoscopy was performed on June 24 (day 22). Localized pleural adhesion and diffuse thickening were noted on the pleural surface of the thoracic cavity (Fig. 2a–b). Disappointingly, the pleural biopsy showed nonspecific inflammatory findings. The pathology of the pleura was only reported as chronic, nonspecific inflammation with fibrosis. Acid-fast bacilli, granuloma and caseous necrosis tests were all negative. Additionally, brushing of the intercostal pleura predominantly revealed lymphocytes, fibrohistiocytes, and mesothelial cells without organisms or malignant cells. There were also no notable findings in the pleural tissues.

Although the smears and cultures for tuberculous evidence were negative, given the highly endemic nature of TB in China and the fact that the previous chest CT scan showed fibrotic tissue and calcification, Mycobacterium tuberculosis was considered. Therefore, the patient received empirical anti-TB therapy with oral isoniazid, rifampin, ethambutol and intravenous administration of levofloxacin two days after the thoracoscopy (on June 26 (day 24)). On the following day, the patient occasionally complained of headache, dizziness, vomiting and night sweats. His temperature was even higher after taking the anti-TB therapy, ranging from 38.2 to 39.5 °C. A lumbar puncture was ordered, but the patient refused. A brain CT (on June 30 (day 28)) only showed several lacunar infarcts in the right basal ganglia. A blood sample for the cryptococcal antigen (CrAg) test was also obtained and sent to Huashan Hospital, Fudan University, Shanghai, P.R. China.

On July 4 (day 32), the patient’s condition deteriorated. His temperature was elevated to 40.2 °C, and he had a severe headache and fainted in the ward. He was immediately transferred to the respiratory intensive care unit (RICU) immediately. His oxygen saturation had dropped to 70 %, and ABG analysis results were pH 7.456, PaCO_2_ 32.0 mmHg, and PaO_2_ 56.3 mmHg. The patient accepted non-invasive ventilation and other support for 2 days. A lumbar puncture was also performed immediately, and the cerebrospinal opening pressure was 400 mm H_2_O (Table 3). *Cryptococcus neoformans* was detected in the cerebrospinal fluid (CSF) (Fig. 2c), and the relevant parameters are shown in Table 3. The anti-tuberculous agents and immunosuppressives were stopped, and intravenous hydration and antifungal therapy were administered. The patient was treated with amphotericin B liposome, 5-flucytosine and voriconazole without interruption. On July 7 (day 35), the CrAg latex agglutination titres in the serum were as high as 1:1280. Furthermore, the serum cultures were reported twice positive for *C. neoformans* on July 11 (day 39), one week after the lumbar puncture. However, the pleural fluid and CSF cultures were all negative for bacterial organisms, mycobacteria and fungi. The serum Candida antigen test was also negative, and the β-D-glucan level was negative, at 4.5 pg/ml (normal < 20 pg/ml).

**Table 3:** Cerebrospinal fluid parameters

Date:	July 4	July 18	August 4
Colour:	clear	slight turbid	clear
WBCs (/μl)	0	70	9
RBCs (/μl)	0	370	7
Pandy’s test	slightly positive	slightly positive	negative
Pressure (mm H_2_O)	≥400	280	280
Protein (mg/L)	553	244	631
Glucose (mmol/L)	<1.11	1.9	3.5
Chlorides (mmol/L)	109	111	118
Smear/Count (/μl)			
*Cryptococcus neoformans*	(+)/580	(+)/1600	(+)/410

*WBC* white blood cell, *RBC* red blood cell

Interestingly, follow-up of the pathological analysis of the pleural biopsy revealed small yeast forms surrounded by a clear halo, as determined using periodic acid-Schiff stain (PAS) and methenamine silver stain (Fig. 2d–e). Upon careful review of the previous pleural histological sections (haematoxylin-eosin (HE) stained) (Fig. 2f), a pathologist found Cryptococcus-like organisms that had been previously overlooked. Thus, pleural cryptococcosis was the cause of the pleural effusion.

Disseminated cryptococcosis consisting of pleural cryptococcosis, cryptococcal meningitis, and cryptococcal septicaemia, was diagnosed. Considering the immunosuppressed state of the patient and the diagnosis of disseminated cryptococcosis with cryptococcal septicaemia, the patient was treated with amphotericin B liposome, 5-flucytosine and voriconazole for the first 11 days, then amphotericin B liposome combined with 5-flucytosine sustained to 8 weeks, which was then changed to fluconazole for maintenance. The left pleural effusion no longer accumulated, and the fever, headache and dry cough subsided. The patient improved and became asymptomatic three weeks after the antifungal therapy. A follow-up chest CT (Fig. 1e–h) revealed resolution of the infiltrate.

## Discussion and conclusion

*C.neoformans* is a nonmycelial, budding, encapsulated, opportunistic, pathogenic, and yeast-like fungus. Inhalation of either the yeast form or basidiospores from an environmental source, such as bird droppings or soil, is believed to cause infection with this opportunistic fungus [8]. The organism may remain confined to the lungs but also frequently disseminates to the CNS, along with the bones, skin, and other parts of the body, depending on the host’s immune status [9]. The most commonly recognized form of human infection due to *C. neoformans* is meningitis, but the fungus typically establishes infection throughout the respiratory tract [10].

The clinical presentation of cryptococcal disease is highly variable and occasionally consists of atypical manifestations. Cryptococcal disease can present as a cough, sputum, shortness of breath, mild fever, headache, chest pain and other symptoms, whereas nearly one-third of pulmonary cryptococcosis cases were discovered by chest X-ray due to a lack of symptoms. Disseminated disease can occur in any organ, but the CNS is the most vulnerable. CNS disease specifically presents with headache, dizziness and other neurological symptoms.

Reeves first described pleural effusion as a manifestation of cryptococcosis in 1941 [11]. Newman et al. reported the first case of cryptococcal pleural effusion in association with acquired immune deficiency syndrome (AIDS) [3]. With a declining incidence of AIDS-related cryptococcosis in the highly active antiretroviral therapy (HAART) era and with increasing use of immunosuppressives worldwide, non-human immunodeficiency virus (HIV)-infected individuals (with or without predisposing factors) may become the predominant group affected by cryptococcosis [12]. In Rozenbaum’s research, only one patient manifested with pleural effusion among 11 patients who had cryptococcosis along with other diseases or the use of immunosuppressive drugs [13]. According to a review by Chao-Chuang Chou, pleural effusion presented in only one patient among 14 patients studied in Taiwan [14]. Here, we report the first case of pleural effusion as the initial clinical presentation in a renal transplant recipient who was diagnosed with fungaemia and disseminated cryptococcosis. By reviewing and summarizing the characteristics and oversights of the clinical course, we hope to contribute to understanding of the disease and to aid in reducing misdiagnoses. Early targeted treatment for cryptococcal disease and intensive care may ultimately improve poor prognoses.

Pleural effusion has rarely been reported as the initial clinical presentation in cryptococcosis. To our knowledge, only 4 cases have been reported in the English-language literature to date [4–7]. Cryptococcosis is difficult to diagnose and may be overlooked, especially when pleural effusion is the only clinical manifestation.

In the case described here, pathology consultation was used to identify the aetiology of the pleural effusion. Additional analyses via PAS and methenamine silver staining revealed numerous Cryptococcus organisms in pleural sections, providing reliable evidence of cryptococcal pleuritis. Regrettably, after reviewing previous HE staining, we found Cryptococcus-like organisms (unstained capsules) that had been overlooked in the initial examination. In this case, the atypical manifestation of the pleural effusion, along with the macroscopic observation and pathologiacl study, caused the early-stage misdiagnosis. In reference to cases reported in the literature, multiple plaques and nodules have been found on the pleural surface in most cases, and pathological findings of cryptococcal pleuritis have shown multiple yeast-like fungal bodies in the inflammatory granuloma [15].

Pleural effusion cultures for Cryptococcus are usually negative, which make diagnosis even more difficult. Young et al. [7] reviewed 26 cases of cryptococcal pleural effusions based in English-language literature, and only 12 patients (42 %) had cultures positive for pleural effusion. The negative culture results may have been due to the small number of fungi in the pleural fluid. In Salyer’s opinion, Cryptococcus can survive in the pleura in the form of a granuloma that releases a large number of antigens into pleural effusion, which is consistent with his research [16]. Similar results in our case support this theory, as the pleural effusion cultures were negative, but the serum was positive for cryptococcal antigens. It has been postulated that the release of antigen, rather than organism growth, is responsible for pleural manifestations. In particular, various clinical manifestations (pleural effusions and others) have been described in HIV-infected patients due to acquired immune deficiency [17]. In the present case, pleural effusion was observed as an unusual initial clinical presentation in a transplant recipient with cryptococcosis. This manifestation is not typical of cryptococcal infection but probably occurred because our patient was on immunosuppressive therapy. His total CD4+ cell count was about 200/ul, less than HIV-infected patients whose CD4+ cell count were about 250/μl.

A cryptococcosis diagnosis is usually made by demonstration of the presence of the organism using histopathology, culture or detection of cryptococcal antigens in the blood and CSF, and by CrAg testing of the pleural fluid. Detection of the capsular antigen is a reliable diagnostic method, and identifying Cryptococcus via histopathological analysis is the gold standard for confirming cryptococcosis. Organisms, and especially yeasts, which are only weakly stained with HE stain, may be difficult to find in routine sections. Gomori-methenamine silver stains is a characteristic black colour, and PAS stains the yeast wall a red colour. According to the literature, the detection rates of *C. neoformans* with these two stains are 100 % [18]. India ink staining is the traditional method for identifying Cryptococcus by delineating the refractile mucinous capsule surrounding the organism, giving the organism a characteristic “starry night” appearance. However, the sensitivity and specificity of India ink staining can be highly variable and are often operator-dependent.

Approximately 8 % of invasive fungal infections in solid-organ transplant (SOT) recipients are due to cryptococcosis [19]. The overall incidence of cryptococcosis in SOT recipients is approximately 2.8 % and ranges from 0.3 to 5 %, with a mortality rate of 20 to 40 % [20, 21]. Once the CNS is involved, the mortality rate is as high as 50 %. Early diagnosis and treatment are crucial to improve the survival of patients with cryptococcosis, and especially disseminated cryptococcosis. In the case described here, the patient received amphotericin B liposome combined with 5-flucytosine and voriconazole for the first 11 days, then amphotericin B liposome combined with 5-flucytosine sustained to 8 weeks, after which this regimen was changed to fluconazole for maintenance. The patient was still on treatment and being followed up at the time that this report was written.

The current guidelines from the Infectious Diseases Society of America (IDSA) recommend treating SOT recipients with disseminated, CNS, or severe pulmonary cryptococcosis with at least 2 weeks of induction therapy with a combination of amphotericin B lipid complex (ABLC) and 5-flucytosine [22]. This regimen slightly differs from Chinese expert consensus on the strategies for treating cryptococcosis. The recommendation from Chinese experts for the management of CNS and pulmonary cryptococcosis in HIV-negative patients is induction therapy with ABLC combined with 5-flucytosine for a minimum of 8 weeks. This difference might be partly attributable to disparities between gradually increasing the dose up to effective dose (which takes time) and administrating lower maintaining doses of ABLC in Chinese [23].

Voriconazole, which is a synthetic derivative of fluconazole, is a relatively new broad-spectrum triazole with excellent in vitro activity against *C. neoformans* (voriconazole exhibits superior in vitro activity compared with fluconazole), good penetration of the blood–brain barrier, and extended activity against moulds and yeasts that were not susceptible to earlier azole-type drugs [24–28]. In fact, several case studies have reported the successful treatment of cryptococcal meningitis with voriconazole [26–28]. The IDSA prefers to recommend amphotericin B deoxycholate (AmBd) plus flucytosine for induction therapy, rather than triple therapy (amphotericin B, flucytosine, and fluconazole), based on data from a randomized trial by Brouwer et al. [22]. Chinese experts suggest that triple therapy (AmBd, flucytosine and voriconazole) could be considered in cryptococcal meningitis patients with AIDS [23]. We chose triple therapy (ABLC, flucytosine, and voriconazole) for the first 2 weeks in this patient. Because it usually cost nearly 2 weeks for gradually increasing the dose up to the effective dose of ABLC, voriconazole could be an important complementary drug during this period. But the patient’s liver function turned bad during triple therapy, we have to stop the voriconazole on the 12th day of antifungal therapy, triple therapy only sustained 11 days, then amphotericin B liposome combined with 5-flucytosine sustained to 8 weeks. As we know, cryptococcal meningitis is fatal, even with specific treatment, so helping patients to survive an acute attack and stabilizing their condition for further consolidation therapy are vital. In this case, we chose triple therapy (ABLC, flucytosine, and voriconazole) for the first stage of induction therapy, with a composite evaluation of safety and efficacy and achievement of a good curative effect. Reassuringly, the patient’s temperature returned to normal, his pleural effusion was absorbed, and his general condition was good. His cerebrospinal pressure decreased to normal, and Cryptococcus were no longer found in the CSF.

*C. neoformans*-associated immune reconstitution inflammatory syndrome (c-IRIS) was initially observed in HIV-infected patients receiving HAART along with rapid reversal of immune deficiency (rapid lymphocyte CD4+ cell count recovery) [29]. The biologic basis of c-IRIS in SOT recipients is believed to be reversal from a Th2 (anti-inflammatory) to a Th1 (pro-inflammatory) cytokine profile [30–32]. The clinical presentation of c-IRIS may be similar to that of *C. neoformans* meningitis. However, c-IRIS has to be differentiated from progression of the cryptococcal disease, although both phenomena can appear simultaneously [22].

The rhG-CSF is known primarily as a haematopoietic growth factor that stimulates the production and release of neutrophilic granulocytes from the bone marrow to limit infection [33]. In our case, this immunosuppressed patient was only treated with rhG-CSF once on day 8. The patient’s condition deteriorated on day 32. rhG-CSF is used to restore neutrophil counts and to prime neutrophil functions, but not lymphocyte counts and functions, to protect vulnerable patients from infection in the clinic [34]. Only one researcher “Hartung” reported continuous rhG-CSF administration could increases monocyte and lymphocyte production, intermittent rhG-CSF treatment could not induce lymphocytosis in volunteers [35, 36]. In this case, followed up of Lymphocytes(/μl) count showed no increasing of Lymphocytes and CD4+ cell after usage of rhG-CSF. The feature of c-IRIS in patients with cryptococcosis is CSF negative fungal culture [1]. In this case, the finding of a positive fungal culture excluded c-IRIS and supported the diagnosis of progression of the cryptococcal disease. Therefore, c-IRIS should not be suspected because of using rhG-CSF in this case. The relationships between rhG-CSF therapy and c-IRIS will need to be further scrutinized.

Cryptococcal infection must be considered in patients with pleural effusion, and especially immunosuppressed patients, even if culture of the pleural fluid is negative. Detection of cryptococcal capsular antigen in the serum and the CSF will be useful in this context. Employing a variety of reliable diagnostic tools, such as a bronchoscopy with bronchoalveolar lavage, video-assisted thoracoscopy, transbronchial biopsy, and exploratory thoracotomy to acquire specimens in the early stages, are also helpful for a definitive diagnosis. Careful review of multiple stained biopsy sections and close communication between the pathologist and the clinician are both important as well.

## Consent

Written informed consent was obtained from the patient for publication of this case. A copy of the written consent is available for review by the Editor of this journal.

## Abbreviations

ADA: Adenosine deaminase
ABLC: Amphotericin B lipid complex
AmBd: Amphotericin B deoxycholate
ABG: Arterial blood gas
AIDS: Acquired immune deficiency syndrome
ANA: Antinuclear antigen
ANCA: Antineutrophil cytoplasmic antibody
c-IRIS: C. neoformans-associated immune reconstitution inflammatory syndrome
*C. neoforman*s: *Cryptococcus neoformans*
CT: Computed tomography
CSF: Cerebrospinal fluid
CrAg: Cryptococcal antigen
CNS: Central nervous system
CRP: C-reactive protein
ESR: Erythrocyte sedimentation rate
FK506: Tacrolimus
HIV: Human immunodeficiency virus
HAART: Highly active antiretroviral therapy
HB: Haemoglobin
HE: Haematoxylin-eosin
IDSA: Infectious diseases society of America
IGRA: Interferon gamma release assay
L: Lymphocyte
LDH: Lactic dehydrogenase
MMF: Mycophenolate mofetil
MP: Methylprednisolone
N: Neutrophil
PCT: Procalcitonin
PCR: Polymerase chain reaction
PLT: Blood platelet
PAS: Periodic acid-Schiff stain
rhG-CSF: Recombinant human granulocyte colony-stimulating factor
RICU: Respiratory intensive care unit
RBC: Red blood cell
SOT: Solid-organ transplant
TB: Tuberculosis
WBC: White blood cell
